# Asian Perspective on Hepatitis B Virus and Hepatitis C Virus Elimination

**DOI:** 10.3390/v17010034

**Published:** 2024-12-29

**Authors:** Apichat Kaewdech, Phunchai Charatcharoenwitthaya, Teerha Piratvisuth

**Affiliations:** 1 Gastroenterology and Hepatology Unit, Division of Internal Medicine, Faculty of Medicine, Prince of Songkla University, Songkhla 90110, Thailand; apichat.ka@psu.ac.th; 2 Division of Gastroenterology, Department of Medicine, Faculty of Medicine Siriraj Hospital, Mahidol University, Bangkok 10700, Thailand; phunchai@yahoo.com; 3 NKC Institute of Gastroenterology and Hepatology, Songklanagarind Hospital, Faculty of Medicine, Prince of Songkla University, Songkhla 90110, Thailand

**Keywords:** HBV, HCV, elimination, prevention, treatment, Asia

## Abstract

Hepatitis B virus (HBV) and hepatitis C virus (HCV) infections remain significant public health challenges in Asia, affecting millions and contributing to substantial morbidity and mortality. The prevalence of these infections varies across the region, with factors such as vaccination coverage, healthcare infrastructure, and sociocultural barriers influencing the epidemiology of both viruses. The persistent burden of chronic HBV, particularly in older populations, and the evolving HCV genotype landscape highlight the need for targeted, region-specific strategies. Universal screening programs have emerged as essential tools for detecting undiagnosed cases and optimizing healthcare resource allocation. Given the overlapping epidemiology of HBV and HCV, comprehensive public health interventions tailored to the unique contexts of different Asian countries are crucial for achieving global elimination goals. This review examines the epidemiological trends, challenges, and opportunities for addressing HBV and HCV in Asia, emphasizing the importance of overcoming sociocultural barriers to improve prevention, diagnosis, and treatment efforts across diverse populations.

## 1. Introduction

Hepatitis B virus (HBV) and hepatitis C virus (HCV) pose significant public health challenges in Asia, affecting millions of individuals and contributing to substantial morbidity and mortality. HBV, a hepatotropic DNA virus, has impacted nearly one-third of the global population, with an estimated 254 million people chronically infected as of 2022 [1]. This figure surpasses the global burden of other infectious diseases, such as HIV, tuberculosis, and malaria [2,3]. Recognizing its impact, the World Health Organization (WHO) has set ambitious goals to reduce new HBV infections by 90% and HBV-related deaths by 65% by 2030 through a comprehensive approach involving vaccination, improved diagnostics, and expanded treatment access [4,5].

The prevalence of HBV across Asia is highly variable. Some regions, like East Asia, report prevalence rates exceeding 10%, while others have significantly lower rates [6]. Similarly, HCV prevalence trends vary across the region [7]. These differences emphasize the need for targeted approaches to address the complexity of viral hepatitis in Asia and work toward eliminating HBV and HCV according to global health targets. Universal screening programs have proven to be both cost-effective and essential for optimal healthcare resource allocation, underscoring their importance in the fight against these infections [8].

This review provides a comprehensive overview of the public health importance of HBV and HCV in Asia, comparing regional epidemiology with global trends. It also discusses strategies for prevention, recent advances in antiviral therapies, and treatment accessibility across different Asian regions. Lastly, the review explores the challenges of achieving widespread treatment coverage and addresses barriers to both national and regional elimination efforts.

## 2. Epidemiology

The epidemiology of HBV and HCV in Asia is shaped by several factors, including geographic location, awareness of transmission and prevention, and lifestyle differences [9]. Countries with a higher burden of HBV and HCV often face challenges such as inadequate healthcare infrastructure and insufficient public knowledge about hepatitis prevention [10]. Moreover, variations in study methodologies and population characteristics complicate the understanding of the true burden of these infections [10], which calls for public health strategies that are tailored to the diverse demographic and geographic contexts of the region.

### 2.1. Hepatitis B Virus Epidemiology

The prevalence of HBV infection across Asia demonstrates significant regional variation. In the Indian subcontinent, the overall prevalence is estimated at approximately 3%, with specific countries’ reporting rates as follows: Pakistan at 6%, Bangladesh at 5%, India at 3%, and Nepal at 1% [11]. In Southeast Asia, the WHO estimates that around 40 million people are living with chronic HBV infection [12]. In China, the overall HBV prevalence has been around 3% in recent years, with rural areas exhibiting higher infection rates compared to urban regions [13]. A meta-analysis has shown that the eastern provinces of China have a higher prevalence of HBV, which may be influenced by migration patterns within the country [13]. National surveys in China revealed fluctuations in the HBsAg carrier rate: it was 8.75% in 1979, increased to 9.75% in 1992, and decreased to 7.18% by 2006 [14]. By 2006, an estimated 93 million individuals in China were living with chronic HBV infection [14]. In Tibet, the prevalence of chronic HBV infection in children younger than 14 years old has tended to decrease over time, likely due to the slow and late increase in birth dose vaccination compared to other countries in Asia [15]. In Turkey, the introduction of universal HBV vaccination in 1998 has led to a decline in prevalence among younger populations, though older individuals born before the vaccine’s implementation remain at a higher risk [16].

Occult HBV infection plays a significant role in the transmission of the virus and can impact the clinical outcomes of affected individuals. A meta-analysis estimated the prevalence of occult HBV infection at approximately 4% across Asia, with certain populations, such as those living with HIV, experiencing even higher rates [17]. Subgroup analysis by region revealed a prevalence of 3% in East Asia, 9% in West Asia, 3% in South Asia, and 9% in Southeast Asia [17]. These findings highlight the persistent public health challenge posed by HBV in Asia and underscore the need for strengthened vaccination and screening programs to curb its spread.

The pooled prevalence of HBV genotypes across countries in Asia is described in Table 1 [18].

### 2.2. Hepatitis C Virus Epidemiology

HCV infection remains a major public health concern in Asia, with considerable variability in prevalence across different countries. India and China are among the nations with the highest burden of HCV, contributing to a substantial proportion of the region’s cases [19]. Estimates suggest that between 49.3 and 64.0 million adults in Asia are anti-HCV positive, with notable prevalence rates in countries like Pakistan (4.7%), and Taiwan (4.4%) [7]. Common risk factors for HCV transmission in the region include healthcare-related infections, unscreened blood transfusions, and injection drug use, which persist in certain areas [7].

HCV genotypes also display regional variations. Genotype 1 is prevalent in Australia and North Asia, while Genotype 3 dominates in India Pakistan, Thailand, and Malaysia. Genotype 4 is commonly found in parts of the Middle East [7]. In Southeast Asia, Genotype 1 accounts for the majority of infections (46.8%), followed by Genotype 3 (23.1%) [20]. The pooled prevalence of HCV genotypes in Asia is summarized in Table 2 [20].

High-risk populations exhibit a significantly higher seroprevalence of HCV. For example, in Northern Vietnam, 89% of people who inject drugs tested positive for HCV RNA, underscoring a serious public health challenge in this group [21]. Similarly, elevated HCV seroprevalence has been reported among other vulnerable populations, including men who have sex with men and sex workers, with rates as high as 28.4% in certain regions of Vietnam [21]. Hemodialysis patients in Vietnam also face an elevated risk, with a reported HCV prevalence of 26%, which is notably higher than in other Asia-Pacific countries [21].

### 2.3. Seroprevalence Trends

The seroprevalence trend of HBV infection in Asia has shown a significant decline over the past few decades, largely due to the success of widespread vaccination programs [16,22]. In China, the prevalence of HBsAg decreased from 9.6% during 1973–1984 to 3% by 2021, with the most pronounced reductions observed in younger age groups [8]. Similar downward trends have been reported in countries such as Vietnam, Mongolia, and Taiwan, where vaccination initiatives have been pivotal in lowering the rates of HBV infection [23]. For example, Thailand has seen the HBsAg seroprevalence rate drop to less than 1.0% among vaccinated individuals [24]. However, challenges persist, particularly in older populations, where seroprevalence rates remain elevated or are increasing [8].

In parallel, global trends in HCV infection have improved since 1990, with significant reductions in age-standardized incidence and mortality rates, although these improvements are not uniform across countries [19]. According to the Global Burden of Disease 2019 report, many Asian nations have seen declines in HCV-related epidemiological indicators [25,26]. In China, for instance, age-standardized HCV rates decreased markedly between 1990 and 2019 [19,21]. Notably, the increase in reported HCV cases in China, from 21,000 annually in 2003 to 210,000 by 2011, is primarily attributed to improved detection and screening efforts rather than a true rise in prevalence [21].

The seroprevalence of HCV across Asia exhibits significant regional variation, coupled with a diverse distribution of genotypes. Historically, Genotype 1b was predominant, but its prevalence has decreased, while Genotype 2a has become more common, particularly in the northern and southern regions [27]. Additionally, Genotype 3 is increasingly prevalent, especially in countries like India and Pakistan [28]. This shift in the genotype landscape highlights the need for enhanced surveillance and tailored public health strategies to effectively manage HCV infections across Asia [29].

## 3. Risk Factors and Transmission Routes

HBV and HCV share common transmission pathways, primarily spreading through contact with infected blood and bodily fluids. This overlap often leads to co-infection in high-risk groups, particularly those exposed to parenteral transmission risks, such as intravenous drug users and individuals engaging in unprotected sexual activity [30,31]. While therapeutic blood product administration and injecting drug use are key transmission routes for HCV in Asia, they account for only 30–60% of anti-HCV-positive cases, depending on the geographical context. Additional transmission routes, including unsafe medical procedures, tattooing, acupuncture, and even sexual contact, further complicate the epidemiology of HCV [31]. In contrast, HBV transmission has been found to correlate closely with socio-economic factors, with higher infection rates observed in areas with lower gross domestic product per capita and higher population density [2].

### 3.1. HBV Transmission

The transmission of HBV in Asia is influenced by several factors, including cultural practices, healthcare access, and the prevalence of risk factors. Vertical transmission from mother to child remains the primary mode of HBV acquisition in many Asian countries, responsible for over 50% of cases in endemic areas [32]. This type of transmission highlights the importance of public health interventions aimed at interrupting perinatal infection [3]. Early-life horizontal transmission is also a significant contributor to HBV spread, particularly among children [33,34]. Close contact with infected family members or caregivers, often through contaminated household surfaces, plays a substantial role in spreading the virus. HBV has the ability to survive on surfaces for extended periods [35], increasing the risk of transmission in domestic settings.

In high-prevalence regions, such as India, intrafamilial horizontal transmission has been identified as a major factor in sustaining HBV infection. A study conducted in eastern India found that intrafamilial transmission surpassed sexual transmission in maintaining the HBV carrier pool within families [36]. Research from northern India further supported this finding, with horizontal transmission accounting for 50% of cases within families of chronic liver disease patients, while vertical transmission accounted for 17% [36]. Among adults, horizontal transmission commonly occurs through sexual contact and injection drug use. Both sexual activity with infected individuals and sharing needles are recognized transmission routes, particularly among men who have sex with men and those with multiple sexual partners [37,38]. Additionally, outbreaks of HBV have occurred in long-term care facilities due to insufficient infection control practices [37].

Approximately half of chronic HBV infections in Asia are acquired perinatally, resulting in chronic HBV infection in around 90% of cases. Therefore, preventing vertical transmission is crucial for reducing the prevalence of chronic HBV infection in Asia. In contrast, horizontal transmission is the common route of HBV infection in adults. However, less than 5% of adults who acquire the infection progress to chronic HBV infection [39].

### 3.2. HCV Transmission

HCV transmission predominantly occurs through direct contact with contaminated blood, including unsafe injection practices, sexual behavior, and intravenous drug use. Genetic analyses of transmission networks have shown that these contact-based routes are the primary drivers of HCV epidemics, especially in Asia [40,41]. Unsafe injection practices remain a major concern in many low-income regions, where the reuse of syringes and poor hygiene conditions during medical procedures are common [19]. Sexual transmission, although less common, is of particular concern among individuals co-infected with HIV, especially within populations of gay and bisexual men [42]. Additionally, in drug-using people, sexual transmission may occur through needle sharing or other high-risk behaviors, complicating efforts to trace and control HCV transmission pathways [40].

Certain regions of Asia have reported alarmingly high HCV prevalence rates among specific at-risk populations. For instance, the HCV seroprevalence among hemodialysis patients in Vietnam is reported at 26%, which is significantly higher than rates observed in other countries within the Asia-Pacific region [21]. Other key populations, such as people who inject drugs and commercial sex workers, also show elevated HCV seroprevalence [21], reflecting the enduring impact of unsafe injection practices and inadequate healthcare infrastructure in these communities [43]. These historical and structural factors contribute to the ongoing transmission of HCV, underscoring the need for targeted interventions in high-risk groups.

## 4. Public Health Strategies

### 4.1. Prevention

To effectively combat HBV and HCV in Asia, a multifaceted approach is essential. Key strategies include establishing robust vaccination programs in underserved areas, raising awareness and improving health literacy among at-risk populations, expanding screening efforts in accordance with national guidelines, and implementing harm reduction initiatives [4,38]. These interventions have demonstrated efficacy in reducing transmission rates and improving health outcomes, supporting the goal of eliminating HBV and HCV as significant public health threats [38,44,45].

#### 4.1.1. Vaccination Programs

One of the most effective tools in preventing HBV transmission is the implementation of widespread vaccination campaigns, particularly targeting at-risk groups. Universal hepatitis B vaccination programs have been pivotal in reducing infection rates. For example, the WHO’s Expanded Program on Immunization, initiated in 1974, aims to ensure universal access to critical vaccines, including the hepatitis B vaccine [38]. Despite these efforts, in some regions, the administration of the birth dose of the HBV vaccine remains suboptimal [46], particularly in rural and impoverished areas where home births are common. Additionally, many infants fail to receive the complete three-dose series due to challenges such as poor vaccine management, inadequate healthcare staffing, and insufficient record-keeping systems [44]. Adolescents and young adults born before the introduction of universal infant vaccination programs are also at risk of HBV infection, as many may have acquired the virus in early childhood and face a heightened risk of developing chronic liver disease later in life [47]. Strengthening vaccination coverage, especially among infants, can substantially reduce the incidence of HBV and its associated complications.

Significant progress has been made in HBV prevention in China through nationwide vaccination initiatives. Since the introduction of the HBV vaccine into routine immunization schedules in 1992, coupled with the removal of vaccine copayments, the country has achieved a marked reduction in chronic HBV infection rates among children [8,13]. By 2015, vaccination coverage for the full three-dose series surpassed 90%, reflecting the success of China’s comprehensive strategy, which includes screening pregnant women and conducting catch-up vaccination campaigns [3,13].

Home deliveries remain not uncommon in some countries in Asia, such as Bangladesh and Myanmar, often involving untrained traditional birth attendants. This may result in low rates of HBV vaccination uptake at birth [48].

#### 4.1.2. Public Awareness Campaigns

A major barrier to addressing HBV and HCV is the lack of public awareness regarding these infections. Globally, fewer than 5% of individuals with chronic hepatitis are aware of their condition [4]. This problem is exacerbated by difficulties in reaching vulnerable populations and the limited knowledge among healthcare providers [4]. Confidentiality concerns surrounding test results further hinder diagnosis and treatment in some regions.

Community-based initiatives aimed at improving awareness and promoting education about HBV and HCV are crucial. Such programs have demonstrated that community engagement is key to overcoming obstacles related to testing, treatment, and vaccination [49]. Successful initiatives often involve collaboration with community leaders, creating care linkages, ensuring program sustainability, and dedicating trained staff [14,50]. One example is the Community-Based Collaborative Innovation (CCI) project in China, which integrates administrative and technical resources to combat hepatitis infections [14]. Additionally, missed opportunities for screening and vaccination by healthcare providers highlight the need for enhanced provider awareness and education, especially for high-risk populations [49].

#### 4.1.3. Screening and Intervention Strategies

Public health efforts to control HBV and HCV in Asia have evolved to include diverse screening and intervention programs. Screening initiatives are most effective when coupled with appropriate follow-up and strong referral networks to ensure that patients receive timely care [38]. Point-of-care and reflex testing can facilitate prompt diagnosis and treatment initiation, helping to contain the spread of both HBV and HCV [38]. Universal screening programs are highly recommended, as they have proven to be both cost-effective and essential for optimizing healthcare resource allocation [8]. Despite some studies reporting HBV screening coverage ranging from 26% to 96%, variability in vaccine uptake emphasizes the need for targeted strategies to enhance access to and effectiveness of vaccination programs [22].

Community involvement plays a vital role in ensuring the success of screening and vaccination campaigns. For example, the HBV and HCV Screening Campaign at the Mahama Refugee Camp in Rwanda demonstrated that engaging local health workers and community representatives facilitated the organization of screening activities and increased participation [22]. Collaborations with local stakeholders were frequently cited as factors that improved the accessibility and cultural sensitivity of these programs, thereby enhancing their overall effectiveness [22].

Efficient referral systems are critical for managing patients diagnosed with HBV or HCV. The CCI project incorporating a two-way referral system allows for the management of mild cases within the community while referring more severe cases to higher-level healthcare facilities [14]. This approach not only improves treatment efficiency but also alleviates the burden on tertiary hospitals. Early treatment of infected individuals helps prevent complications such as liver cirrhosis and failure, leading to better health outcomes and a reduced long-term disease burden [3].

#### 4.1.4. Harm Reduction Strategies

Harm reduction strategies aim to minimize the health risks associated with substance use without requiring abstinence. These public health interventions include syringe exchange programs, the distribution of naloxone to prevent opioid overdoses, and supervised drug consumption sites. These initiatives are crucial in reducing the transmission of infectious diseases, including HBV and HCV, particularly among people who inject drugs. In Asia, an estimated 300,000 people who inject drugs are living with HBV, and approximately 2.6 million are infected with HCV—substantially higher figures than those for HIV [51]. However, despite the significant burden of disease, harm reduction strategies remain limited in many countries, possibly due to a lack of understanding and awareness among patients, incomplete national policies, economic constraints, stigma, and laws criminalizing illicit drug use [52]. Expanding these initiatives is vital for reducing the incidence of HBV and HCV among drug-using populations and for supporting overall public health efforts to eliminate these infections.

### 4.2. Treatment

#### 4.2.1. Advances in Antiviral Therapies for HBV

Currently, there are two treatments available for CHB treatment in Asia, including nucleos(t)ide analogs (NAs) and pegylated interferon-alpha (PEG-IFN-α) [53]. The goal of therapy for CHB infection is to improve the quality of life and survival of the infected person by preventing the progression of the disease to cirrhosis, decompensated cirrhosis, end-stage liver disease, HCC, and death and preventing the transmission of HBV to others [54]. This goal can be achieved if HBV replication can be suppressed in a sustained manner. Increasing evidence from a meta-analysis of seven randomized control trials (RCT) involving 3463 CHB patients with a mean follow-up time of 2 years showed that antiviral therapy significantly decreased the risk of decompensation and cirrhosis but not HCC development and all-cause mortality [55]. Moreover, 35 observational studies involving 59,201 CHB patients with a mean follow-up time of 5 years reported that antiviral therapy decreased the risk of cirrhosis, HCC, and all-cause mortality [55].

#### 4.2.2. Indication for Treatment (Table 3)

Treatment considerably started in pre-cirrhotic CHB patients if they have persistently elevated ALT levels > 2 times the upper limit of normal (ULN) and HBV DNA > 20,000 IU/mL if HBeAg-positive and > 2000 IU/mL if HBeAg-negative [56,57]. The EASL suggested early treatment in patients who have ALT elevation > ULN with significant HBV DNA > 2000 IU/mL [58]. In patients with persistently normal ALT, the non-invasive assessment of liver fibrosis or liver biopsy determining the severity of necroinflammation should be considered. Patients with significant fibrosis or at least moderate necroinflammation should be treated. In cirrhotic patients, most liver societies’ guidelines suggested treatment irrespective of HBV DNA and ALT levels.

In 2020, the expert consensus on CHB treatment was established by several Asian countries, including China, Taiwan, Hong Kong, Japan, and the Republic of Korea. The treatment criteria were defined as follows [59]:

- (a) HBV DNA ≥ 2000 IU/mL and ALT ≥ 1× ULN.
- (b) HBV DNA ≥ 2000 IU/mL, ALT < 1× ULN, with ≥F2 fibrosis and/or ≥A2 necroinflammation.
- (c) Presence of cirrhosis with detectable HBV DNA.
- (d) HBV DNA ≥ 2000 IU/mL, ALT < 1× ULN, accompanied by a family history of cirrhosis or HCC, extrahepatic manifestations, or age > 40 years.

Patients with cirrhosis and/or HCC should be treated regardless of ALT levels if HBV DNA is detectable.

In 2022, the Chinese Society of Hepatology, in collaboration with the Chinese Society of Infectious Diseases, released updated guidelines for CHB prevention and treatment [60]. These guidelines simplified treatment criteria for non-cirrhotic CHB patients with detectable HBV DNA and persistently elevated ALT levels (>ULN) after excluding other causes. The guidelines also expanded indications to include patients with detectable HBV DNA, irrespective of ALT level, who meet any of the following criteria:

- (a) Family history of cirrhosis or HCC.
- (b) Age ≥ 30 years.
- (c) Evidence of significant inflammation (Grade ≥ 2) or fibrosis (Stage ≥ 2).
- (d) Extrahepatic manifestations related to HBV infection.

Notably, the guidelines recommend initiating treatment in HBsAg-positive patients with cirrhosis regardless of HBV DNA level, differing from other guidelines that suggest treatment in cirrhotic patients only when HBV DNA is detectable. All indications are strongly recommended based on moderate-quality evidence.

In March 2024, the WHO proposed expanding the eligibility criteria for HBV treatment [61]. The updated criteria recommend treatment for CHB patients with significant fibrosis, regardless of ALT or HBV DNA levels. Additionally, CHB patients with HBV DNA > 2000 IU/mL and elevated ALT levels above the ULN are eligible for treatment. Treatment is also warranted for patients meeting any of the following criteria:

- Coinfection with HIV, HDV, or HCV.
- Family history of liver cancer or cirrhosis.
- Immunosuppression.
- Presence of comorbidities such as diabetes or metabolic-dysfunction associated steatotic liver disease (MASLD).
- HBV-related extrahepatic manifestations.

**Table 3:** HBV treatment indication.

Guideline	Cirrhosis	Non-Cirrhosis
AASLD [56]	All adults with CHB cirrhosis with detectable HBV DNA	HBV DNA > 20,000 IU/mL for HBeAg (+) and >2000 IU/mL for HBeAg (–) plus either: ALT ≥ 2× ULN at least 3 months Significant histologic disease
EASL [58]	All adults with CHB cirrhosis with detectable HBV DNA	HBV DNA > 2000 IU/mL + ALT >ULN + moderate necroinflammation and/or moderate fibrosis (or liver stiffness > 9 kPa) HBV DNA > 20,000 IU/mL + ALT > 2× ULN
APASL [57]	All adults with CHB cirrhosis with detectable HBV DNA	ALT >2× ULN + HBV DNA > 20,000 IU/mL for HBeAg (+) and >2000 IU/mL for HBeAg (–) ALT 1–2× ULN + HBV DNA > 20,000 IU/mL for HBeAg (+) and >2000 IU/mL for HBeAg (–), and biopsy shows moderate to severe inflammation or significant fibrosis
THASL [62]	All adults with CHB cirrhosis with detectable HBV DNA	HBV DNA at least 2000 IU/mL plus either: ALT > 1.5× ULN at least 3 months Significant histologic disease
China [60]	All adults with CHB cirrhosis regardless of HBV DNA levels	Detectable HBV DNA plus ALT >ULN or Detectable HBV DNA regardless of ALT level, who meet any of the following criteria: Family history of cirrhosis or HCC Age of at least 30 years Evidence of significant liver inflammation (Grade ≥ 2) or fibrosis (Stage ≥ 2) Presence of extrahepatic manifestations related to HBV infection
WHO [61]	All adults with CHB cirrhosis with detectable HBV DNA	Significant fibrosis, regardless of ALT or HBV DNA or HBV DNA > 2000 IU/mL + ALT > ULN or The presence of any one of the following criteria: coinfection with other viruses, family history of liver cancer or cirrhosis, immunosuppression status, having comorbidities such as diabetes, MASLD, HBV extrahepatic manifestations

AASLD, American Association for the Study of Liver Diseases; ALT, Alanine Aminotransferase; APASL, Asian Pacific Association for the Study of the Liver; CHB, Chronic Hepatitis B; EASL, European Association for the Study of the Liver; HBeAg, Hepatitis B e Antigen; HBV, Hepatitis B Virus; HCC, Hepatocellular Carcinoma; kPa, Kilopascal; MASLD, Metabolic-dysfunction Associated Steatotic Liver Disease; THASL, Thai Association for the Study of the Liver; ULN, Upper Limit of Normal; WHO, World Health Organization.

The two therapeutic approaches available for the suppression of HBV replication include antiviral agents (NAs) and immune-based therapies with interferon α (IFN-α) or pegylated interferon α (PEG-IFN-α).

#### 4.2.3. NAs

NAs inhibit HBV reverse transcription via RNA polymerase. The preferred oral antiviral agents suggested by most liver societies are high-efficacy drugs and high resistance barriers including entecavir (ETV), tenofovir disoproxil fumarate (TDF), and tenofovir alafenamide (TAF) [56,57,58]. Most guidelines suggest ETV or TAF in patients over age 60 or who have comorbidities with renal or bone disease. In almost CHB, long-term NAs therapy can achieve virological remission, but indefinite therapy may be required owing to the low chance of HBsAg clearance. Moreover, the longer duration of treatment associated with side effects, cost of drugs, and drug adherence [63]. Treatment discontinuation in selected patients could be beneficial in terms of an increased chance of HBsAg loss, a finite treatment duration, and minimized long-term side effects, particularly bone and renal impairment from TDF [63]. However, about 30% of patients might develop clinical relapse after NA discontinuation [64,65,66,67]. HBV biomarkers, including HBsAg level, hepatitis B core-related antigen (HBcrAg), and HBV RNA, could help identify those who would benefit most from stopping NAs [64,65].

#### 4.2.4. PEG-IFN-α

PEG-IFN-α has the advantage of a finite duration without the possibility of drug resistance. This drug has immunomodulatory effects, enhancing cell-mediated immune response and antiviral properties as a result of blocking viral transcription, degrading pgRNA, inhibiting translation, and modifying proteins in the viral replication process [68]. Epigenetic modifications of cccDNA and the degradation of minichromosomes by the activation of APOBEC3s are important additional mechanisms of PEG-IFN-α for eliminating HBV [69]. PEG-IFN-α therapy for 1 year results in HBeAg seroconversion in 22–27% and HBsAg loss in 3–5% of HBeAg-positive patients compared to 22% HBeAg loss and 0–3% HBsAg loss after the 1-year treatment of NAs [70,71,72,73,74,75,76]. Despite the higher HBsAg loss, the drawbacks of PEG-IFN-α are the requirement of subcutaneous injection, significant adverse effects that need frequent clinical and laboratory monitoring, and contraindication in certain patients, i.e., decompensated cirrhosis, severe exacerbation of hepatitis, and autoimmune or psychiatric illnesses. Identifying the patients who are likely to respond to PEG-IFN-α can optimize therapy using qHBsAg while under treatment with PEG-IFN-α. Patients who have HBsAg < 1500 IU/mL or HBsAg decline ≥ 10 at week 12 of treatment are likely to respond to treatment. Therefore, motivating patients to complete 48-week treatment is considered. Stopping PEG-IFN-α treatment or switching to the other treatment strategy in patients who meet the stopping rule (HBsAg level > 20,000 IU/mL or lack of any HBsAg decline at Week 12 of treatment in HBeAg-positive and HBeAg-negative patients, respectively) [53]. Identifying non-responders could allow the patients to withdraw from the treatment early and avoid the side effects of switching to other treatment strategies early.

#### 4.2.5. Novel Therapy

There are two classes of novel therapy for HBV that directly target HBV replication with DAAs and enhance immune response via either innate immunity or adaptive immune response. Direct-acting antivirals (DAAs) target various stages of the HBV replication cycle, including the following:

Entry Inhibitors: Bulevirtide, for instance, impedes HBV from entering hepatocytes. It has shown promise in clinical trials, particularly for hepatitis delta virus (HDV) co-infection, and has received conditional approval in the European countries [77].

Capsid Assembly Modulators (CAMs): These compounds disrupt the formation of the viral capsid, which is essential for HBV replication. Agents like ABI-4334 are currently undergoing Phase 1b clinical trials to assess their efficacy and safety [78].

#### 4.2.6. HBV RNA Inhibitors

Small Interfering RNAs (siRNAs): These molecules degrade HBV RNA, reducing viral protein production. For example, JNJ-3989 has demonstrated efficacy in reducing viral load in Phase 2b studies [79].

Antisense Oligonucleotides (ASOs): ASOs bind to HBV RNA, preventing its translation into viral proteins [80].

RNA Binding Protein Inhibitors: These agents interfere with proteins that stabilize HBV RNA, leading to its degradation [81].

Immune Modulators: These therapies aim to enhance the body’s immune response against HBV by stimulating innate or adaptive immunity [53]. Approaches include toll-like receptor agonists and therapeutic vaccines designed to boost HBV-specific immune responses [82].

While these novel therapies are in various stages of clinical development and have shown encouraging results, they are not yet approved for general clinical use. Ongoing research and clinical trials are essential to establish their safety and efficacy profiles.

#### 4.2.7. Advancements in HCV Treatment

The advent of DAAs has revolutionized HCV treatment globally, including in Asia [83]. These agents offer high cure rates, shorter treatment durations, and improved safety profiles compared to previous interferon-based therapies [84,85,86]. Approval of pan-genotypic regimens: Medications such as sofosbuvir/velpatasvir [87] and glecaprevir/pibrentasvir [88] have been approved in several Asian countries, providing effective treatment options across all HCV genotypes. In Thailand, the approved treatment for all patients with active viremia from chronic hepatitis C infection is a 12-week regimen of sofosbuvir/velpatasvir [89]. For non-cirrhotic patients, this combination is administered without ribavirin, while for cirrhotic patients, ribavirin is included in the treatment protocol. Holybuvir is a novel pan-genotypic hepatitis C virus NS5B inhibitor currently under development in China [90]. This agent has the potential to improve the efficacy of hepatitis C treatment.

#### 4.2.8. Challenges in HBV and HCV Treatment

Despite therapeutic advancements, several challenges impede the elimination of HBV and HCV in Asia [91]. A recent WHO report found that in the Southeast Asia region, only 2.8% of CHB patients had been diagnosed, and just 0.1% had received treatment, compared with a global diagnosis rate of 13.4% and a treatment rate of 2.6% [1]. The specific challenges include the following:

- Limited access to antiviral therapies: High costs and regulatory hurdles restrict the availability of novel therapies in low- and middle-income countries, leading to disparities in treatment access [92]. The cost of HCV treatment could become more affordable in many economically constrained countries due to the possibility of transferring licenses from brand manufacturers to generic manufacturers through the Medicines Patent Pool.
- Late diagnosis: A significant proportion of individuals remain undiagnosed due to inadequate screening programs, resulting in delayed treatment initiation and increased transmission risk [1].
- Stigmatization: Social stigma associated with HBV and HCV infections discourages individuals from seeking testing and treatment, perpetuating the cycle of transmission [93,94].

### 4.3. Regional Initiatives and Guidelines

**Figure 1:**
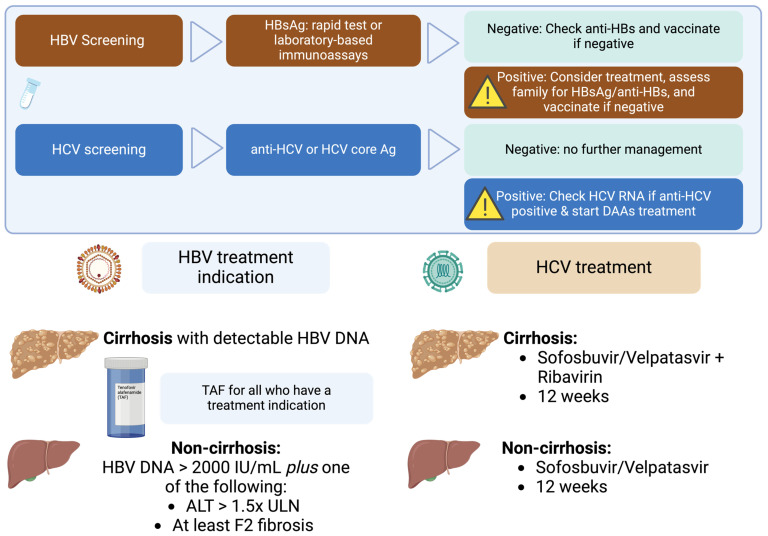
Algorithm for HBV and HCV Screening, Diagnosis, and Treatment Guidance in Thailand.

Several Asian countries have implemented national strategies to combat HBV and HCV:

- China: The “Action Plan for Elimination of HCV in China (2021–2030)” aims to enhance screening, improve treatment access, and reduce transmission rates [95].
- Japan: The government has established comprehensive screening programs and subsidized treatment costs to encourage early diagnosis and therapy [96].
- India: The National Viral Hepatitis Control Program focuses on increasing awareness, expanding screening, and providing free treatment to affected individuals [97].
- Thailand: The policymakers provide a more convenient algorithm for HBV and HCV treatment guidance, as shown in Figure 1.

- anti-HBs, Antibodies to Hepatitis B Surface Antigen; anti-HCV, Antibodies to Hepatitis C Virus; DAAs, Direct-Acting Antivirals; HBsAg, Hepatitis B Surface Antigen; HBV, Hepatitis B Virus; HCV, Hepatitis C Virus; HCV core Ag, Hepatitis C Virus Core Antigen; RNA, Ribonucleic Acid; TAF, Tenofovir Alafenamide.

### 4.4. Future Directions

To achieve the WHO’s goal of eliminating HBV and HCV as public health threats by 2030, Asian countries must address existing challenges through the following:

- Scaling up screening programs: Implementing widespread, accessible screening initiatives to identify and treat infected individuals promptly [98].
- Enhancing treatment access: Negotiating with pharmaceutical companies to reduce antiviral prices and facilitating the approval of generic formulations [99,100].
- Public education campaigns: Raising awareness about HBV and HCV transmission, prevention, and the benefits of early treatment to reduce stigma and encourage healthcare engagement [101].
- Integration of services: Incorporating HBV and HCV testing and treatment into existing healthcare services, such as primary care and harm reduction programs, to streamline patient care [102].

## 5. Conclusions

Eliminating HBV and HCV as public health threats in Asia is achievable with targeted, region-specific strategies. Despite progress through vaccination and advanced antiviral treatments, challenges persist due to limited healthcare infrastructure, social stigma, and high treatment costs. Expanding screening programs, especially among high-risk populations, and strengthening healthcare systems to support affordable treatment access are essential. Public health campaigns to raise awareness and reduce stigma, coupled with collaborative efforts aligned with WHO guidelines, are critical. By integrating vaccination, harm reduction, and community engagement, Asia can advance toward hepatitis elimination and relieve future generations from these infections.

## Figures and Tables

**Table 1:** Pooled prevalence of HBV subtypes and high-prevalence countries in Asia.

HBV Subtypes	Pooled Prevalence (%)	95% CI	Countries with High Prevalence of More than 30%
A1	26.7	20.9–33.6	Bangladesh, Nepal, Afghanistan
A5	25.6	20.1–32.1	
B1	27.6	21.1–35.3	Indonesia, Vietnam, China, Taiwan, Sri Lanka
B2	29.5	24.7–34.7	
B3	29.9	22.5–38.5	
B4	28.6	21.9–36.4	
B5	28.1	21.4–35.9	
B7	26.3	20.3–33.3	
B8	25.8	20.1–32.5	
C1	33.5	27.0–40.7	Japan, Bangladesh, Thailand, China, Malaysia, Korea, Afghanistan, Cambodia, Lebanon
C2	40.0	33.3–47.0	
C3	27.0	20.6–34.6	
C4	26.7	20.4–34.0	
C5	27.9	21.4–35.4	
C6	26.1	20.1–33.2	
C7	26.0	20.2–32.8	
C8	26.4	20.4–33.4	
C9	28.2	21.4–36.2	
C10	26.1	20.1–33.1	
C17	26.1	20.1–33.1	
D1	32.5	24.7–41.4	Iran, Uzbekistan, Pakistan, Tajikistan, India, Turkey, Azerbaijan, Jordan, Serbia, Nepal, Oman, Yemen, Afghanistan
D2	33.1	25.3–41.9	
D3	26.0	20.2–32.9	
D4	26.0	20.1–33.0	
D5	26.1	20.2–33.0	
D6	26.0	20.2–32.9	
D-DEL	26.0	20.2–32.7	
E	0.6	0.4–0.9	
F	0.6	0.3–1.2	
H	0.6	0.4–0.8	
I	0.7	0.5–1.0	

CI, confidence interval; HBV, hepatitis B virus.

**Table 2:** Pooled prevalence of HCV genotypes in Asia.

HCV Genotypes	Pooled Prevalence (%)	95% CI
1	46.8	43.2–50.4
2	4.6	3.5–5.9
3	23.1	19.4–27.2
4	1.1	0.7–1.5
5	0.8	0.4–1.3
6	16.5	13.8–19.6

CI, confidence interval; HCV, hepatitis C virus.
